# Epitope similarity cannot explain the pre-formed T cell immunity towards structural SARS-CoV-2 proteins

**DOI:** 10.1038/s41598-020-75972-z

**Published:** 2020-11-04

**Authors:** Ulrik Stervbo, Sven Rahmann, Toralf Roch, Timm H. Westhoff, Nina Babel

**Affiliations:** 1 Center for Translational Medicine, University Hospital Marien Hospital Herne, Ruhr-University, Bochum, Germany; 2 Berlin-Brandenburg Center for Regenerative Therapies, and Institute of Medical Immunology, Charité – Universitätsmedizin Berlin, Corporate Member of Freie Universität Berlin, Humboldt-Universität Zu Berlin, Berlin Institute of Health, Berlin, Germany; 3 Genome Informatics, Institute of Human Genetics, University of Duisburg-Essen, Duisburg, Germany

**Keywords:** Viral infection, Protein sequence analyses, MHC

## Abstract

The current pandemic is caused by the SARS-CoV-2 virus and large progress in understanding the pathology of the virus has been made since its emergence in late 2019. Several reports indicate short lasting immunity against endemic coronaviruses, which contrasts studies showing that biobanked venous blood contains T cells reactive to SARS-CoV-2 S-protein even before the outbreak in Wuhan. This suggests a preformed T cell memory towards structural proteins in individuals not exposed to SARS-CoV-2. Given the similarity of SARS-CoV-2 to other members of the Coronaviridae family, the endemic coronaviruses appear likely candidates to generate this T cell memory. However, given the apparent poor immunological memory created by the endemic coronaviruses, immunity against other common pathogens might offer an alternative explanation. Here, we utilize a combination of epitope prediction and similarity to common human pathogens to identify potential sources of the SARS-CoV-2 T cell memory. Although beta-coronaviruses are the most likely candidates to explain the pre-existing SARS-CoV-2 reactive T cells in uninfected individuals, the SARS-CoV-2 epitopes with the highest similarity to those from beta-coronaviruses are confined to replication associated proteins—not the host interacting S-protein. Thus, our study suggests that the observed SARS-CoV-2 pre-formed immunity to structural proteins is not driven by near-identical epitopes.

## Introduction

The current coronavirus disease 2019 (COVID-19) pandemic is caused by the severe acute respiratory syndrome coronavirus-2 (SARS-CoV-2)^1^ with devastating consequences. SARS-CoV-2 infections have a broad spectrum of manifestations, ranging from asymptomatic to severe pneumonia and acute respiratory distress syndrome^2^. The reason for this broad range is still unclear, but markedly decreased immune cell numbers^3,4^, together with cytokine storm^5^, and dysregulation of lung infiltrating immune cells^6–9^ have been associated with critical COVID-19 manifestations. The more severe virulence of SARS-CoV-2 is in contrast to the four endemic coronaviruses OC43, HKU1, 229E, and NL63, which are responsible for the common cold with usually mild symptoms^10^.

Coronaviruses are single positive stranded RNA viruses and with a genome size of about 30 kb are the largest of the known RNA-viruses. The SARS-CoV-2 genome contains 14 open reading frames (ORFs), where the ORF10 is not translated into protein^11^. In addition to a number of non-structural proteins, the ORFs translates into the four structural proteins: spike (S) glycoprotein, small envelope (E) glycoprotein, membrane (M) glycoprotein, and the nucleocapsid (N) protein. The most non-structural proteins are encoded by ORF1a and ORF1b, where ORF1a is translated into the polyprotein pp1a and through a slippery sequence near the end of ORF1a the translation is continued into the ORF1b which produce the 7096 amino acid long polyprotein pp1ab^12^. The autoproteolytic cleavage of the polyproteins pp1a and pp1ab creates the non-structural proteins, which form the complex replicase machinery. Among these are the essential RNA-dependent RNA polymerase (RdRp) embedded in ORF1b^11^.

T cells recognize peptides presented in the context of the human leukocyte antigen (HLA) class I and class II molecules. Peptides presented on the class I HLAs are generally recognized by CD8^+^ cytotoxic T cells, while CD4^+^ T helper cells recognize peptides bound on the HLA class II molecule. T cells are known to be cross reactive^13,14^, that is, a single T cell can recognize similar peptides derived from different pathogens presented by HLA molecules. Additionally, T cells are known to be promiscuous and can recognize many different epitopes^15^.

The SARS-CoV-2 virus elicits a T cells response during the infection^6,16–18^. However, mounting evidence suggests that 20–50% of unexposed individuals are capable of responding to peptides derived from the S-, N-, and M-proteins of the SARS-CoV-2 virus^6,16–24^, indicating a pre-existing immunity to these SARS-CoV-2 proteins. A single influenza epitope has been identified by comparison of T cell receptors^25^, but the original pathogenic source of this pre-formed T cell memory is generally unclear. Interestingly, identical SARS-CoV-2 specific T cell receptors were observed in multiple convalescent donors^17^ indicating a similar source of the pre-formed immunity. There is also some evidence of pre-existing SARS-CoV-2 antibodies^26^, which like for the T cells indicate that previous infections can cause SARS-CoV-2 cross-reactive immunity.

Given the high sequence similarity, endemic coronaviruses have been suggested likely inducers of the observed pre-existing immunity although other sources are possible^27^. However, immunity to these coronaviruses appears short-lived as antibody titers return to baseline levels at 4–12 months after infection^28–30^. Importantly, it can also not be excluded that re-infection with the same coronavirus type can occur within a single year^31^. Both SARS-CoV-1, which caused the 2002–2004 SARS epidemic, and MERS-CoV, which emerged in 2012, have both been shown to elicit a long lasting T cell memory^20,32^. In contrast, the T cell memory towards the endemic coronaviruses is not clear. For influenza it is clear that recurrent infections and epidemics are due to the accumulation of mutations in the hemagglutinin and neuraminidase^33^. However, the genetic drift of endemic coronaviruses seems to be considerably slower than for Influenza A and B^34,35^. Collectively, this indicates that other frequently encountered pathogens besides the endemic coronaviruses could have generated the preexisting immunity.

In the present report, we evaluate commonly occurring human pathogens for epitopes with a very high similarity to potential SARS-CoV-2 epitopes.

## Results

**Figure 1:**
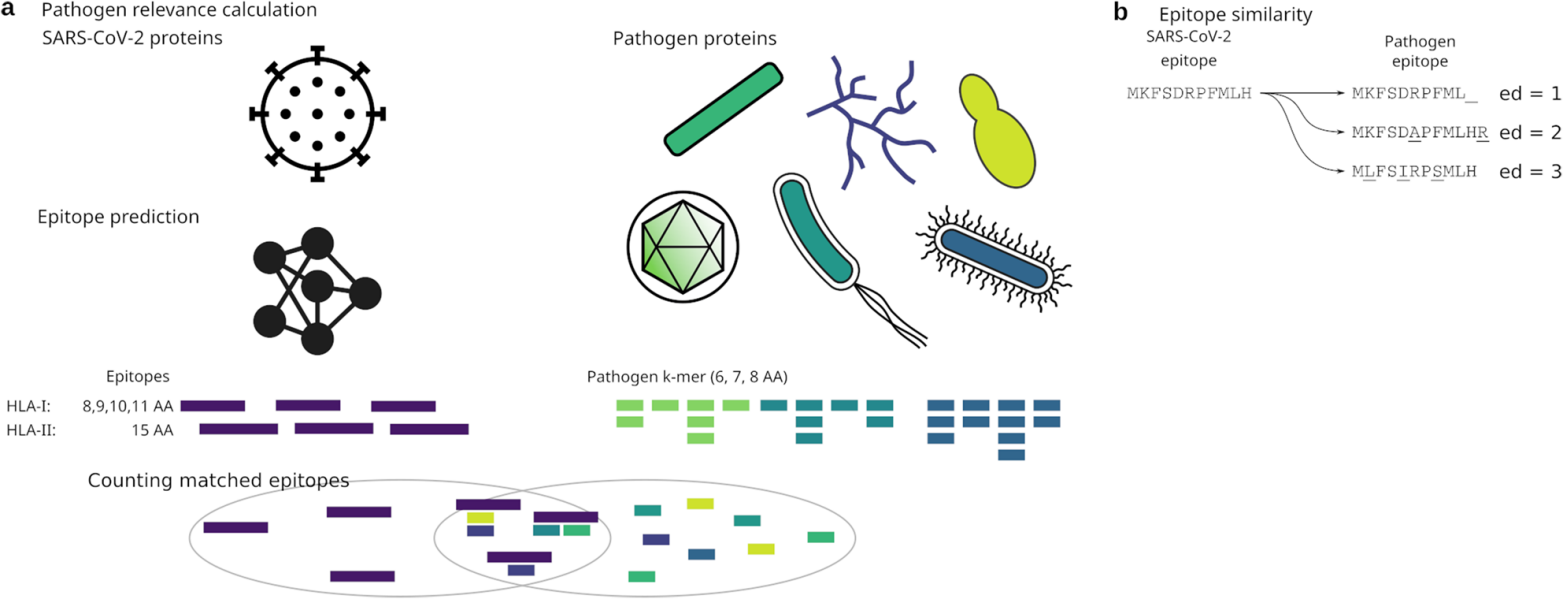
Analysis approach. (**a**) *k*-mers for *k* ϵ {6, 7, 8} were extracted from the proteins of relevant human pathogens and compared to epitopes predicted in the SARS-CoV-2 proteins. The epitope prediction was by netMHCpan and netMHCIIpan. Pathogens were ranked based on exact *k*-mer hits to the epitopes. (**b**) Principle of edit distance determination of epitopes. The SARS-CoV-2 epitope MKFSDRPFMLH has a edit distance of 1 when compared to the putative pathogen epitope MKFSDRPFML_ because of the missing histidine at the C-terminus. The distance between MKFSDRPFMLH and MKFSDAPFMLHR is 2 because of the D6A exchange and the additional arginine at the C-terminus. The K2L, D5I, and F8S exchanges give rise to an edit distance of 3 between MKFSDRPFMLH and MLFSIRPSMLH.

We identified a number of pathogens commonly causing infections in the European population (Supplementary Tables 2–5). The list of pathogens included 32 viruses, 11 fungi, 26 bacteria and 2 parasites. We obtained all protein sequences for these pathogens from NCBI, and compared these to predicted HLA-I and HLA-II binding epitopes in SARS-CoV-2, and ranked the pathogens based on a relevance score (see Methods; Fig. 1a) based on short exact sequence matches of length *k* ("*k*-mers"). We limited the analysis to include only the five most common HLA alleles in the European population as reported in the Allele Frequency Net Database^36^ (Supplementary Tables 6–7).

We observed different relevance scores for the same pathogen, depending on the length of *k*-mers (Supplementary Figs. 1 and 2). For *k* = 6 we found that all viruses were in the upper half when ranking the relevance scores, while the viruses were in the lower half for *k* = 8. This was independent of the HLA-class epitopes (Supplementary Fig. 1 and 2).

**Figure 2:**
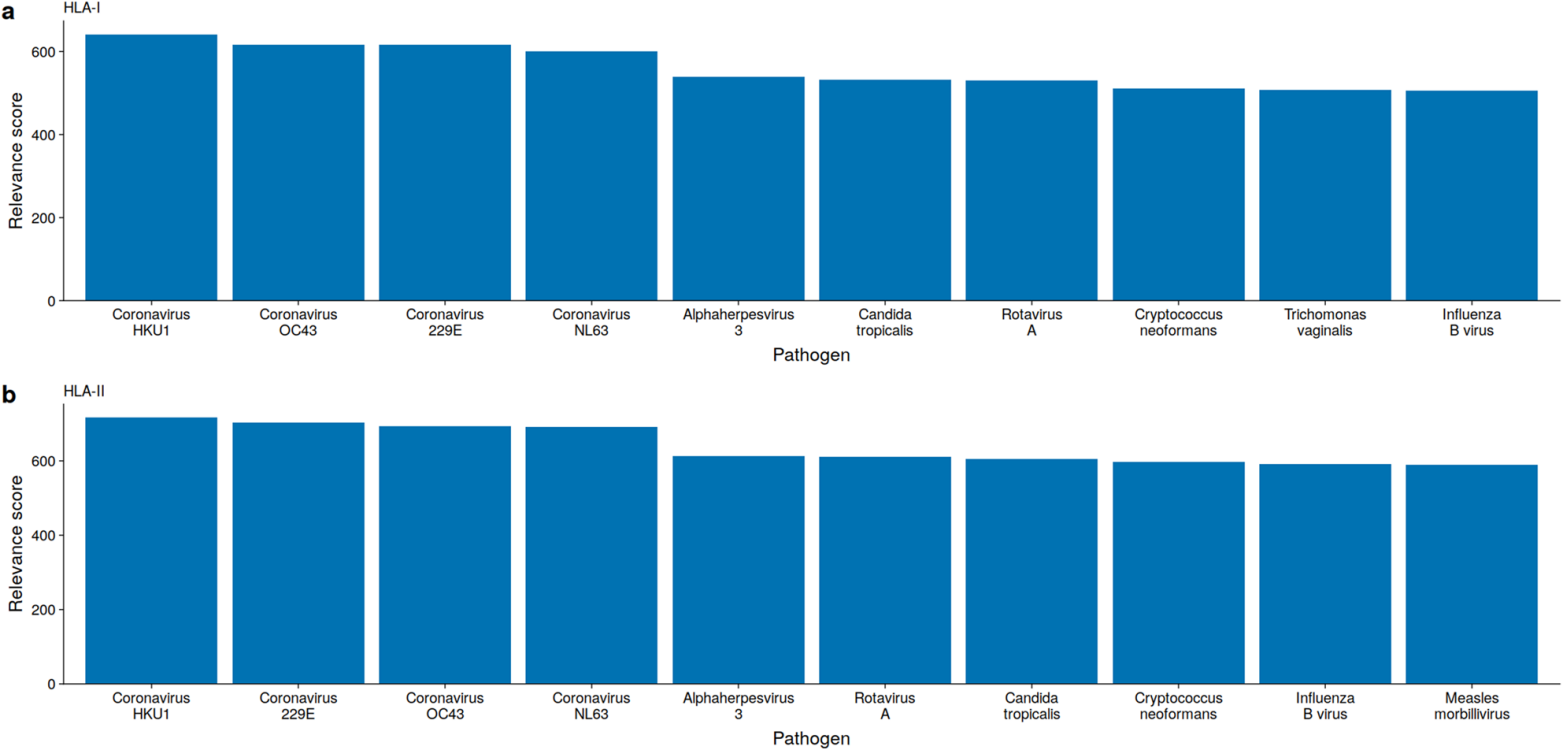
Some viruses and bacteria have peptides (*k*-mers) matching SARS-CoV-2 epitopes. The top 10 pathogen relevance scores were averaged over *k* = 6,7,8 amino acids for (**a**) HLA-I, and (**b**) HLA-II. Pathogen relevance score for each pathogen and *k* are presented in Supplementary Figs. 1 and 2.

Given the overall similarity between coronaviruses, the endemic coronaviruses are expected to have the highest relevance score. Given the compact genome size and highly optimized proteins^37^ it seems likely that only short stretches will have an exact match, even when using a reduced alphabet. Conversely, more complex organisms have larger genome, why the probability of matching longer stretches increases. We therefore focus on pathogens with short (*k* = 6) matches.

Within the top 10 ranking pathogens for HLA-I binding SARS-CoV-2 epitopes based on *k* = 6, we find the two fungi *Candida tropicalis* and *Cryptococcus neoformans*, and the parasite *Trichomonas vaginalis*. Apart from the endemic coronaviruses HKU1, OC43, 229E, and NL63 we find the double-stranded DNA virus *Human alphaherpesvirus 3* (*varicella-zoster virus*, VZV), the double-stranded RNA virus *Rotavirus A* (RV), and the single-stranded negative RNA virus *Influenza B* (Fig. 2a). When scoring the relevance based on matches to predict HLA-II SARS-CoV-2 epitopes, we find a similar result, the only difference being the appearance of *Human Gammaherpesvirus 4* (*Epstein-Barr virus*, EBV) in place of *Trichomonas vaginalis* (Fig. 2b).

Viral genes can be expressed at different times during an infection^38^. However, during the multiplication phase of the virus, the viral products are expressed in excess. To avoid selection of pathogens based on highly similar, but rarely or poorly expressed proteins, we therefore focus on the viruses in the following analysis.

Using netMHCpan and netMHCIIpan we predicted the epitopes in the reference sequences for the coronaviruses OC43, HKU1, 229E, and NL63, as well as *Influenza B*, EBV, RV, and VZV. Assessing the similarity to SARS-CoV-2 predicted epitopes, we calculated the edit distance from each SARS-CoV-2 epitope to each of the predicted epitopes in the selected pathogens (Fig. 1b). The edit distance – also known as the Levenshtein distance – accounts for addition, deletion, and substitution of amino acids to transform one amino acid sequence into another. We opted for this distance metric to allow differences in epitope lengths. The edit distance was calculated per analyzed HLA.

**Figure 3:**
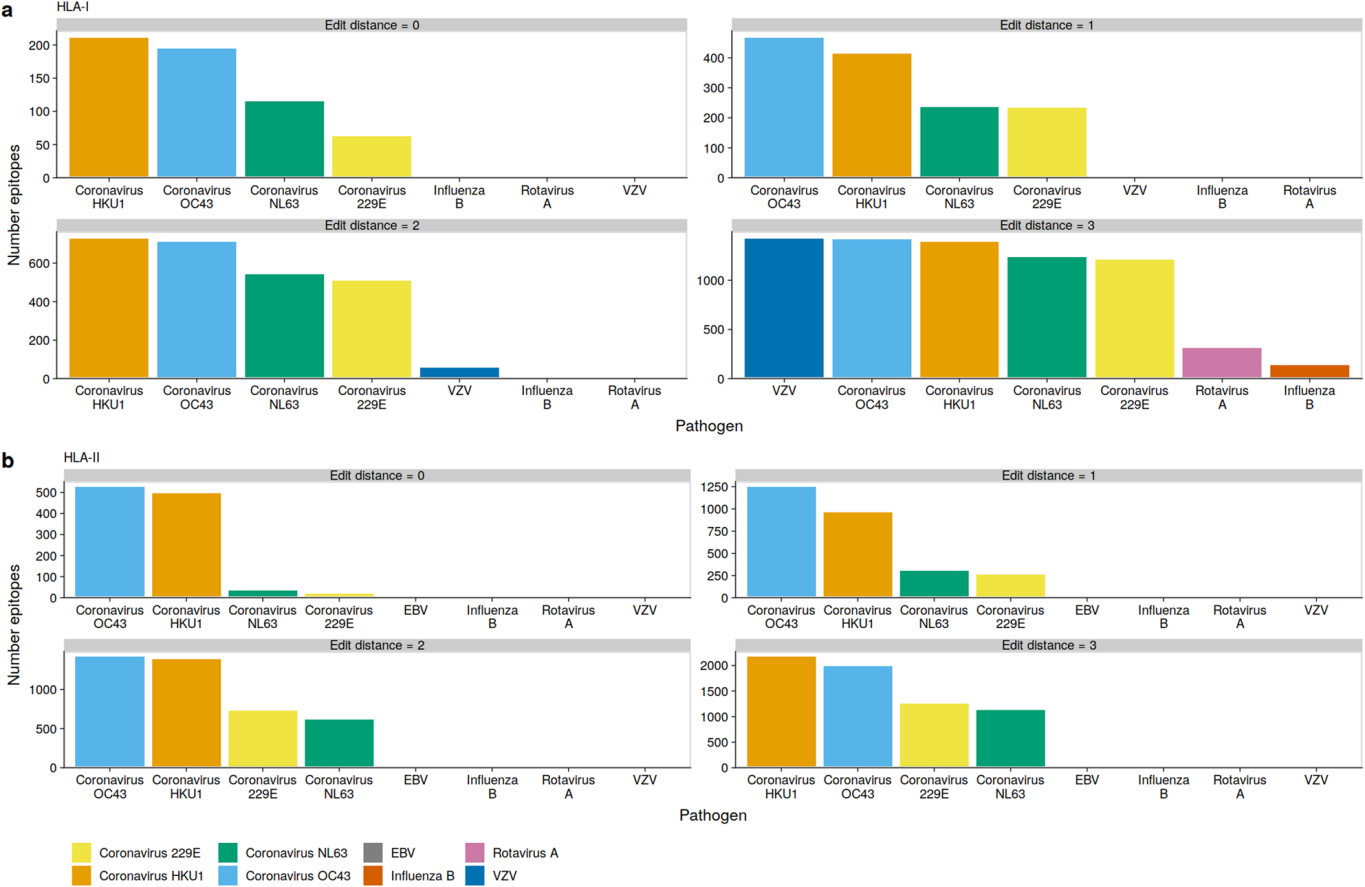
OC43 and HKU1 epitopes can be presented on many HLAs. Epitopes were predicted using netMHCpan and netMHCIIpan in selected pathogens. The similarity between each SARS-CoV-2 epitope and pathogen epitope was calculated using the edit distance, and the number of shortest matches was enumerated. (**a**) Total number of HLA-I epitopes with a edit distance between 0 and 3. (**b**) Total number of HLA-II epitopes with a edit distance between 0 and 3. The pathogens are ordered per plot from highest to lowest, while the fill color is preserved.

We found that the beta-coronaviruses OC43 and HKU1 had the highest number of epitopes identical to the predicted SARS-CoV-2 epitopes. For HLA-I bound epitopes we found 211 and 195 identical epitopes in HKU1 and OC3, respectively (Fig. 3a). For HLA-II bound epitopes we found 493 and 464 identical epitopes in OC43 and HKU1, respectively (Fig. 3b). When the similarity threshold was relaxed to an edit distance of 1 or 2 we found a similar pattern (Fig. 3). Interestingly, if we accept an edit distance of 3 we find the highest number of similar SARS-CoV-2 HLA-I epitopes in VZV, followed by OC43, and HKU1 with 1292, 1189, and 1163 epitopes, respectively (Fig. 3a). This was not reflected in HLA-II bound epitopes. The strong occurrence of SARS-CoV-2 similar VZV epitopes was mainly driven by epitopes on HLA-B and HLA-C, and to a minor degree on HLA-A (Supplementary Figs. 3–5). In agreement with previous reports^39^, we found a large number of identical or similar epitopes between SARS-CoV-1 and SARS-CoV-2 (Supplementary Fig. 6).

We next asked which coronavirus proteins might be the most likely inducers of cross reactivity. Arguably, the epitopes most likely to elicit a cross-reactive response are those found in many corona viruses and are presented by many HLAs. The latter constraint is important, since the current studies demonstrating cross-reactivity do not distinguish HLAs^6,16,18–23^. We first enumerated the number of SARS-CoV-2 epitopes for each of the viral proteins (Supplementary Fig. 7). Given that the OC43 and HKU1 coronavirus strains appear the most likely pathogen to create SARS-CoV-2 reactive T cells, we focused the analysis on these strains.

We found that only epitopes from the SARS-CoV-2 polyprotein pp1ab have identical amino acid sequences to epitopes identified in both OC43 and HKU1. Interestingly, the epitopes from the S-protein are different at three positions or more compared to the amino acid sequences for the predicted OC43 and HKU1 epitopes. Since not only the number of epitopes but also the probability of the epitope to be presented, we also enumerated the number of epitope-presenting HLAs (Supplementary Fig. 8). The highest number of possible HLAs is 15 for HLA-I and 80 for HLA-II (50 DPA1-DPB1 combinations, 25 DQA1-DQB1 combinations, and 5 DRB1). Again, we found the highest number of HLAs and the highest similarity in epitopes from the SARS-CoV-2 polyprotein pp1ab.

**Figure 4:**
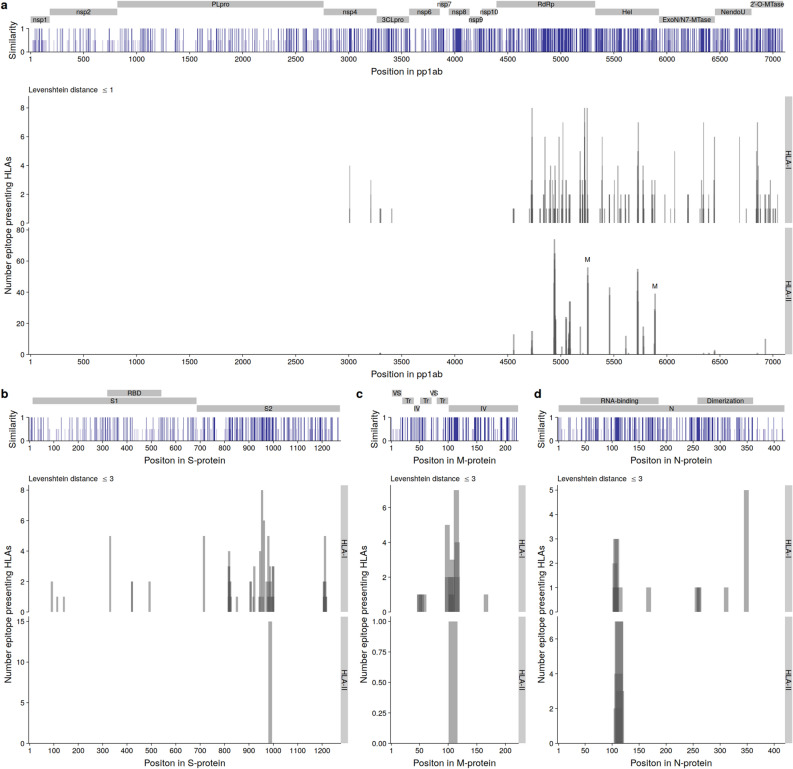
SARS-CoV-2 epitopes from conserved regions are nearly identical to OC43 and HKU1 epitopes. (**a**) Upper panel: Protein sequence for the polyprotein pp1ab from SARS-CoV-2, OC43, and HKU1 were aligned and the similarity between OC43 and HKU1 amino acids to SARS-CoV-2 was calculated. The individual proteins are marked above the similarity graph. Lower panel: The number of HLA-alleles that present SARS-CoV-2 pp1ab epitopes with a edit distance of 1 or less to epitopes predicted in both OC43 and HKU1. Two epitopes previously identified as cross reactive^24^ are marked by ‘M’. (**b**) Upper panel: Protein sequence for the S-protein from SARS-CoV-2, OC43, and HKU1 were aligned and the similarity between OC43 and HKU1 amino acids to SARS-CoV-2 was calculated. The individual proteins are marked above the similarity graph, where RBD gives the receptor binding domain. Lower panel: The number of HLA-alleles presenting SARS-CoV-2 S-protein epitopes with an edit distance of 3 or less to epitopes predicted in both OC43 and HKU1. (**c**) Upper panel: Protein sequence for the M-protein from SARS-CoV-2, OC43, and HKU1 were aligned and the similarity between OC43 and HKU1 amino acids to SARS-CoV-2 was calculated. The individual proteins are marked above the similarity graph: ‘VS’ indicates the portion of the M-protein on the virion surface, ‘Tr’ the transmembrane region, and ‘IV’ the intraviron portion. Lower panel: The number of HLA-alleles presenting SARS-CoV-2 M-protein epitopes with an edit distance of 3 or less to epitopes predicted in both OC43 and HKU1. (**d**) Upper panel: Protein sequence for the N-protein from SARS-CoV-2, OC43, and HKU1 were aligned and the similarity between OC43 and HKU1 amino acids to SARS-CoV-2 was calculated. The individual domains of the N-protein are marked above the similarity graph. Lower panel: The number of HLA-alleles presenting SARS-CoV-2 N-protein epitopes with an edit distance of 3 or less to epitopes predicted in both OC43 and HKU1. The height and color of the similarity graphs designate similarity such that white bars indicate no similarity, light blue bars with half height indicate 50% similarity and dark blue bars with full height indicate 100% similarity. The width of the bar in the lower panels indicates the length of the epitopes. Darker regions indicate overlapping epitopes.

The polyprotein pp1ab is 7096 amino acids long, and 15 nonstructural proteins are created through autoproteolytic cleavage^12^. Comparison of the pp1ab amino acid sequence from SARS-CoV-2, HKU1, and OC43 revealed that the RNA-dependent RNA polymerase (RdRp), the helicase (Hel), the 3′–5′ exoribonuclease (ExoN), and the 2′-O-ribose methyltransferase generally have the highest similarity (Fig. 4a; upper panel). It is also in these regions that the near identical epitopes, as determined by an edit distance of 1 or less, are found (Fig. 4a; lower panel). An experimental study evaluated a set of 117 epitopes form HKU1 and OC43 for their cross reactive potential^24^. Two epitopes from HKU1 OC43 were identified as capable of raising a T cell response, and were nearly identical to two SARS-CoV-2, marked with ‘M’ in Fig. 4a. The S-protein epitopes previously found to expand public T cell clonotypes^17^, were not found to be shared with HKU1 or OC43.

T cell immunity to the structural proteins has received substantial attention^6,16,18–24^. The S2 portion of the S-protein, which constitutes the stalk of the host interacting receptor^12^, shares the highest similarity between SARS-CoV-2, HKU1, and OC43 (Fig. 4b; upper panel). Interestingly, we found some similar HLA-I epitopes (edit distance of 3) but only one HLA-II epitope (Fig. 4b; lower panel). The majority of the HLA-I epitopes and the single HLA-II epitope fall within the relatively conserved portion of the S2. The similarity between SARS–CoV–2, HKU1, and OC43 for the M- and N-proteins is most prominent around position 110 in either of the proteins (Fig. 4c,d; upper panel). This corresponds to the N-terminus of the long intraviron tail of the M-protein, and a small part of the RNA-binding domain of the N-protein. Similar to the observation for the S-protein, we find some similar HLA-I epitopes (edit distance of 3) but only one a single position of HLA-II epitopes (Fig. 4c,d; lower panel). These epitopes also appear in the conserved regions. Collectively, these data indicate that near identical epitopes derived from endemic coronaviruses cannot explain the observed cross-reactivity to structural SARS-CoV-2 proteins.

## Discussion

There is mounting evidence for preformed immunity against the novel coronavirus SARS-CoV-2^16,19–22^. Reports on memory T cell response to the endemic coronaviruses are lacking, but since antibody titers appear transient and frequent re-infections cannot be excluded^28–31^, it is probable that the endemic coronaviruses with low virulence do not create lasting immunity. This then begs the question, which pathogen can generate a memory T cell response cross-reactive to SARS-CoV-2. Through in silico analysis of predicted SARS-CoV-2 epitopes and common pathogens we address this question.

When allowing some dissimilarity, VZV has the highest number of SARS-CoV-2 like epitopes. However, this we only find for HLA-I, and in particular HLA-B and HLA-C, but not for HLA-II. Since SARS-CoV-2 specific T cells are found among both the HLA-I reactive CD8^+^ and among the HLA-II reactive CD4^+^ it is not likely that VZV is the pathogen behind the SARS-CoV-2 cross reactive T cells. Rather, only the endemic coronaviruses, and in particular the beta-coronaviruses, have the highest number of epitopes similar to those predicted in the SARS-CoV-2 virus. However, the similarity between epitopes from SARS-CoV-2 and endemic coronaviruses was concentrated in replication related proteins. Thus, it appears unlikely that endemic coronaviruses should give rise to the observed preformed T cell immunity towards the S-protein. This notion is supported by experimental findings, where cross-reactivity to S-protein is rare^24,40^. A set of near-identical SARS-CoV-2 epitopes have been identified in Mycobacterium bovis^27^ which, in its attenuated form, is used as vaccine against tuberculosis. Although the efficacy of this vaccine against SARS-CoV-2 remains unclear^41,42^, the findings indicate nonetheless that SARS-CoV-2 cross-reactive T cells can have sources widely different from viral pathogens.

It appears that bat coronaviruses require an intermediate host to attain the potential of becoming a human pathogen^10^. For 229E these intermediate hosts are likely camelids while SARS-CoV-1, SARS-CoV-2, and MERS-CoV find intermediate zoonotic hosts in civets, dog or pangolin, and camels, respectively^10,43^. The intermediate host for NL63 is not known. In this context it is interesting that OC43 and HKU1 are the most likely candidates, as the natural host of these viruses are rodents^43^. The effect of these hosts on the pathogenicity of the coronavirus remains a source of debate. However, the immune systems of dogs, camels, pangolins, rodents, and humans have marked differences—murine B cells for instance express the pattern recognition receptor TLR4, which is absent in human B cells^44^. Because of these differences in the immune system, it is unlikely that the same evasive strategy can be applied in different species.

The lack of general epidemiological interest in the endemic coronaviruses render information on the prevalence in Europe elusive. Data collected by the Karolinska Institute in Stockholm, Sweden between 2010 and 2017, and by the West of Scotland Specialist Virology Centre, Greater Glasgow and Clyde, Scotland between 2005 and 2017, show seasonal variation of the four viruses. The peak season is between December and April where 2–4% of patients with respiratory disease were positive for any of the four viruses^45,46^. Though the data is obtained among patients with respiratory diseases, it can be expected to be a reasonable proxy for the prevalence in the greater population.

While the prediction of HLA-I epitopes has high accuracy, the accuracy of HLA-II epitope prediction is lower than that for HLA-I^47^. One reason for this lower accuracy of HLA-II epitope prediction is the dimer structure of the HLA-II making the peptide binding groove. In fact, the HLA-II ɑβ-chain combinations have been estimated to be over 4000 in number. In contrast, the combination of the HLA-I and β2-microglobulin chains yields less combinatorial variation on the receptor. The epitope prediction with netMHCpan depends on a proper training set. By focusing on the most frequent European HLAs, inaccuracies in the epitope prediction are reduced or even avoided. We did not utilize epitope databases like VDJDB^48^ and IEDB^49^, the reason being that these databases have a natural bias towards laboratory model antigen. For instance, the most frequent epitopes in the Immune Epitope Database are human auto antigen and epitopes derived from *Trypanosoma cruzi*.

The amino acid set was reduced from the 20 standard amino acids to 15 amino acids by combining molecules with similar hydrophobic side chains. The advantage is the ease of sequence comparison without alignment since the *k*-mer is a complete substring of the target protein. While the reduced amino acid alphabet is derived from structural considerations^50^, it cannot be excluded that alphabet reduction might skew the results. However, given the requirements of anchor amino acids, which are buried within the HLA-molecule, the results should not change.

One limitation to the study is that we do not consider the frequency of pathogens nor the potential expression level of the proteins and accessibility for the immune system. Both arguably have an effect on the likelihood that an epitope can raise a robust immune response. However, both variables can only be assessed with great uncertainty. Additionally, our analysis hone in on pathogens with the largest number of *k*-mers matching SARS-CoV-2 epitopes, which may exclude relevant pathogens producing few but highly immunogenic SARS-CoV-2 similar epitopes. Another limitation to the study is the reliance on sequence similarity; although related epitopes are likely to interact with the same T cell receptor, this is not guaranteed^51^. This is also observed in the report by Mateus et al.^24^ where a peptide with 80% similarity to a SARS-CoV-2 epitope did not raise a T cell response, while a peptide with only 33% similarity did.

The presentation of different epitopes on different HLA-molecules is well known. In this study, we focus on a small handful of HLAs with prevalence in Europe. Since patient HLAs are rarely evaluated, it is not possible to know if the patients with observed SARS-CoV-2 cross-reactivity carry the same epitopes as evaluated here. Given the prevalence in Europe, it is possible that there is an overlap with some studies, but the specifics are essentially unknown. It is therefore important that the HLAs are disclosed in studies evaluating antigen specific cells, in order to focus in silico studies such as this.

In conclusion, epitope similarity between SARS-CoV-2 and other endemic beta-coronaviruses cannot explain the observed SARS-CoV2 pre-formed immunity towards structural proteins. We conjecture that this observation of preformed immunity is likely driven by structurally different peptides.

## Methods

The SARS-CoV2 protein sequences were downloaded from ViralZone (https://viralzone.expasy.org/89966)^52^, accessed May 29, 2020. Uniprot IDs and common names are listed in Supplementary Table 1. The common human pathogens evaluated in this study are listed in Supplementary Tables 2–5. Pathogen protein sequences were extracted from the NCBI "non-redundant" protein database ("nr", version 5, downloaded from ftp://ftp.ncbi.nlm.nih.gov/blast/db/FASTA on May 31, 2020). The extraction was per pathogen name, as stored in the Taxonomy database. For epitope comparison, the protein reference sequences for the coronaviruses OC43, HKU1, 229E, and NL63, and Influenza B, Human Gammaherpesvirus 4, Rotavirus A, and Human alphaherpesvirus 3, were downloaded from https://ftp.ncbi.nlm.nih.gov/refseq/release/viral on 26.Jun.2020. Validated SARS-CoV-1 epitopes were obtained from IEDB (https://www.iedb.org)^49^. Protein segments for SARS-CoV-2 pp1ab (identifier: P0DTD1) and S-protein (identifier: P0DTC2) were obtained from UniProt (https://www.uniprot.org).

All potential MHC class I and class II epitopes in the SARS-CoV2 protein sequences, and selected pathogen reference sequences, were identified using netMHCpan version 4.1 and netMHCIIpan version 4.0, respectively, with default settings^53^. For this step, the evaluated HLAs listed in Supplementary Tables 6 and 7 were selected based on frequency in the European population as reported in the Allele Frequency Net Database^36^; https://www.allelefrequencies.net, June 1, 2020.

The edit distance was calculated as the Levenshtein distance between each predicted SARS-CoV-2 epitope to a predicted epitope in a selected pathogen was calculated. The calculation as per pathogen and HLA and only the best match was used.

Short peptides of length *k (k*-mers) of lengths 6, 7, and 8 were extracted from the proteins of each of the relevant pathogens and counted, for each value of *k* separately Each such multiset of *k*-mers was pre-filtered so that *k*-mers found only once in a pathogen were removed if those *k*-mers constituted a small fraction (1%) or less of all *k*-mers found for the pathogen. The resulting *k*-mers were matched to the *k*-mers from the predicted epitopes for SARS-CoV-2 using a reduced 15 letter amino acid alphabet^50^. This amino acid alphabet allows mismatches of amino acids with similar properties by letting the large hydrophobic amino acids V, L, I, and M be represented by L, the amino acids Y and F, which are hydrophobic with aromatic side chains by F, and the positively charged K and R by K.

The more complex an organism, the more proteins are expressed. This means that the probability of finding *k*-mers matching epitopes from SARS-CoV-2 increases. To overcome this problem we developed a *pathogen relevance score* to be evaluated for each triple of SARS-CoV-2 protein *p*, pathogen species *s* and value of *k* (note that the quantity also depends on the considered set of epitopes, i.e. HLA class I or class II only or combined). We thus define

$$ S_{p,s,k } : = log_{2} \left[  \left(N_{p,s,k} / T_{p,k}  \right) / \left(  \left(P_{s} + C \right) / \left( {15^{k} + C} \right) \right) \right], $$

where $N_{p,s,k}$ is the number of *k*-mer matched epitopes of protein *p* in species *s*, and $T_{p,k} : = \mathop \sum \limits_{s} N_{p,s,k}$ is the number of such epitopes of protein *p* across all species; their ratio $N_{p,s,k} / T_{p,k}$ is viewed in relation to the "richness" of the *k*-mer set of the pathogen species *s*, i.e. $P_{s}$, the number of distinct *k*-mers in pathogen s, divided by the total number of possible k-mers over the reduced alphabet ($15^{k}$). To avoid bias in favor of underrepresented species (very small $P_{s}$), we add a regularizing constant C when computing the fraction of *k*-mers used by the species. Thus the score is a log-observed-vs-expected-ratio indicating whether species *s* matches unproportionally many SARS-CoV-2 epitopes that cannot be explained by its proteome size alone.

To focus on possibly relevant pathogens in general rather than on a single SARS-CoV-2 protein, we also considered an aggregated score

$$ S_{s,k } : = log_{2} \left[  \left(N_{s,k} / T_{k}  \right) / \left(  \left(P_{s} + C \right) / \left( {15^{k} + C} \right) \right) \right], $$

where $N_{s,k}$ is the total number of *k*-mer matched epitopes in species *s*, and $T_{k} : = \mathop \sum \limits_{s} N_{s,k}$ is the number of such epitopes across all species. The pathogens were then ranked according to score (the highest score obtaining rank 1, the lowest score rank *n*, where *n* is the number of considered species). Then the average rank is computed over the different parameter combinations *k* = 6,7,8.

## Supplementary information

Supplementary Information

## Data Availability

The protein sequences used in this study are available from public sources: SARS-CoV-2 sequences: https://viralzone.expasy.org/89966, NCBI "non-redundant" protein database, version 5: ftp://ftp.ncbi.nlm.nih.gov/blast/db/FASTA, Protein reference sequences: ftp://ftp.ncbi.nlm.nih.gov/refseq/release/viral. The workflow and accompanying Python scripts is available as a Snakefile for use with Snakemake under https://gitlab.com/svenrahmann/corona.
