# Supplementary material for: Epitope similarity cannot explain the pre-formed T cell immunity towards structural SARS-CoV-2 proteins
Source: Sci Rep. 2020 Nov 4;10:18995. doi: 10.1038/s41598-020-75972-z (PMC7642385; doi:10.1038/s41598-020-75972-z)
Supplement: Supplementary file 1 — Supplementary Information [file 41598_2020_75972_MOESM1_ESM.pdf]

Supplement to
Epitope similarity cannot explain the pre-formed T cell immunity towards structural SARS-CoV-2 proteins

Ulrik Stervbo[1,2*], Sven Rahmann[3*], Toralf Roch[1,2], Timm H. Westhoff[1], Nina Babel[1,2]

[1] Center for Translational Medicine, University Hospital Marien Hospital Herne, Ruhr-University Bochum, Germany
[2] Berlin-Brandenburg Center for Regenerative Therapies, and Institute of Medical Immunology, Charité – Universitätsmedizin Berlin, Corporate Member of Freie Universität Berlin, Humboldt-Universität zu Berlin, and Berlin Institute of Health, Berlin, Germany
[3] Genome Informatics, Institute of Human Genetics, University of Duisburg-Essen, Germany

* Equal contribution

Contact:
Ulrik Stervbo: ulrik.stervbo@elisabethgruppe.de
Sven Rahmann: sven.rahmann@uni-due.de

Content

Content 2
Supplementary Methods 3
Workflow and Software 3
Supplementary Tables 4
Supplementary Table 1: SARS-CoV-2 proteins 4
Supplementary Table 2: Selected viruses 5
Supplementary Table 3. Selected fungi 7
Supplementary Table 4. Selected bacteria 8
Supplementary Table 5. Selected parasites 9
Supplement Table 6. Used HLA-I molecules 9
Supplementary Table 7. Used HLA-II molecules 10
Supplementary Figures 11
Supplementary Figure 1: Pathogen relevance score for HLA-I SARS-CoV-2 epitopes 11
Supplementary Figure 2: Pathogen relevance score for HLA-II SARS-CoV-2 epitopes 12
Supplementary Figure 3: Edit distance between HLA-A SARS-CoV-2 epitopes and epitopes in selected pathogen 13
Supplementary Figure 4: Edit distance between HLA-B SARS-CoV-2 epitopes and epitopes in selected pathogen 14
Supplementary Figure 5: Edit distance between HLA-C SARS-CoV-2 epitopes and epitopes in selected pathogen 15
Supplementary Figure 6: Number of known epitopes SARS-CoV-1 shared with predicted SARS-CoV-2 epitopes 16
Supplementary Figure 7: Number of HLA-I and HLA-II presented epitopes in each SARS-CoV-2 protein 17
Supplementary Figure 8: Number of different epitopes presenting HLAs identified for epitope in each SARS-CoV-2 protein. 18
References 19

Supplementary Methods

Workflow and Software

Our workflow is available as a Snakefile for use with Snakemake[1], starting with the download of the "nr" database from NCBI, at gitlab at the URL https://gitlab.com/svenrahmann/corona. The following steps are included, each performed by a specific Python script contained in the above repository.

Required inputs to the workflow are:
- a FASTA file with SARS-CoV-2 protein sequences (sara-cov2.fa),
- a tab-separated file with selected taxIDs of species to consider (species_taxid.tsv),
- a text file with predicted epitopes of SARS-CoV-2 (sars_mhci_peptides.txt, sars_mhcii_peptides.txt; one peptide per line)

All of these are provided in the repository.

The workflow consists of the following steps.
1. Download of the NCBI nr database: Snakefile (rule get_nr)
2. Extract protein sequences of common human pathogens from nr: Snakefile (rule select_species); filterspecies.py
3. Compute statistics on the number of sequences of each selected pathogen present in the nr database: Snakefile (rule count_selected)
4. Extract k-mers from each species' protein sequences for several values of k and for several reduced alphabets: Snakefile (rule get_kmers); getcounters.py
5. Filter out k-mers that only occur once in the set of all proteins of a species for different k, alphabets and filter thresholds: Snakefile (rule filter_kmers): filterkmers.py
6. Create a table of SARS-CoV-2 epitopes by protein with k-mer matches against pathogens. The resulting output files (results/epitopes_{class}_{k}_{alphabet}_{filterthreshold}) have a tab-separated tabular format with 3 columns: (SARS-CoV-2 protein name, pathogen name, peptide sequence of matched SARS-CoV-2 epitope). After all epitopes matched by the same pathogen have been shown, a statistics line (starting with '#') is inserted, giving the pathogen relevance score and other statistical information. At the end of the file, several summary statistics (over all proteins, per pathogen) are given (lines starting with '+'), including overall pathogen relevance scores. The same information is also written into a summary file: Snakefile (rules show_epitopes; summarize_epitopes); showepitopes.py.
7. Compute average pathogen ranks from different values of *k* and filter thresholds, for each epitope class I and II separately and combined, and generate plots of ranked ranks: Snakefile (rule aggregate_results); aggregate.py

Supplementary Tables

Supplementary Table 1: SARS-CoV-2 proteins

Name and function of the SARS-CoV2 proteins obtained from ViralZone[2].

**UniProt ID**	**Common name**
P0DTC1	Replicase polyprotein 1a (pp1a)
P0DTD1	Replicase polyprotein 1ab (pp1ab)
P0DTC2	Spike glycoprotein (S)
P0DTC3	ORF3a protein (NS3a)
P0DTC4	Envelope small membrane protein (E)
P0DTC5	Membrane protein (M)
P0DTC6	ORF6 protein
P0DTC7	ORF7a protein
P0DTD8	ORF7b protein
P0DTC8	ORF8 protein
P0DTC9	Nucleoprotein (N)
P0DTD2	ORF9b protein
P0DTD3	ORF14 protein

Supplementary Table 2: Selected viruses

Names and taxIDs obtained from the NCBI Taxonomy database[3].

Family	Scientific name	TaxID	Taxon level
**ssRNA(+)**			
Caliciviridae	Norwalk virus	11983	Species
Coronaviridae	Human coronavirus OC43	31631	Species
	Human coronavirus HKU1	290028	Species
	Human coronavirus 229E	11137	Species
	Human coronavirus NL63	277944	Species
Matonaviridae	Rubella virus	11041	Species
Picornaviridae	Human rhinovirus A*	147711	Species
	Human rhinovirus B*	147712	Species
	Human rhinovirus C*	463676	Species
	Enterovirus B	138949	Species
	Human hepatitis A virus	208726	Subtype
	Hepatitis B virus	10407	Species
	Hepatitis E virus	12461	no rank
	Hepacivirus C	11103	Species
	Human poliovirus 1	12080	Subtype
	Human poliovirus 2	12083	Subtype
	Human poliovirus 3	12086	Subtype
**ssRNA(-)**			
Orthomyxoviridae	Influenza A virus	11320	Species
	Influenza B virus	11520	Species
Paramyxoviridae	Human respirovirus 1	12730	Species
	Human respirovirus 3	11216	Species
	Human rubulavirus 2	1979160	Species
	Human rubulavirus 4	1979161	Species
	Mumps rubulavirus	1979165	Species
	Measles morbillivirus	11234	Species
Pneumoviridae	Human metapneumovirus	162145	Species
**dsRNA**			
Reoviridae	Rotavirus A	28875	Species
**dsDNA**			
Herpesviridae[1]	Human alphaherpesvirus 1	10298	Species
	Human alphaherpesvirus 3	10335	Species
	Human gammaherpesvirus 4	10376	Species

	Human betaherpesvirus 5	10359	Species
Papillomaviridae	Human papillomavirus	10566	Species

*Subspecies were included by necessity.

[1]Common names: Human alphaherpesvirus 1: Herpes simplex virus type 1; Human alphaherpesvirus 3: Varicella-zoster virus; Human gammaherpesvirus 4: Epstein-Barr virus; Human betaherpesvirus 5: Human cytomegalovirus

Supplementary Table 3. Selected fungi

Names and taxIDs obtained from the NCBI Taxonomy database[3].

Genus	Scientific name	TaxID	Taxon level
**Yeast**			
Candida	Candida albicans	5476	Species
	Candida glabrata	5478	Species
	Candida tropicalis	5482	Species
**Fungus**			
Aspergillus	Aspergillus fumigatus	746128	Species
	Aspergillus flavus	5059	Species
	Aspergillus niger	5061	Species
Cryptococcus	Cryptococcus neoformans	5207	Species
Pneumocystis	Pneumocystis jirovecii	42068	Species
Stachybotrys	Stachybotrys chartarum*	74722	Species
Trichophyton	Trichophyton rubrum	5551	Species
	Trichophyton mentagrophytes	523103	Species

*Subspecies were included by necessity

Supplementary Table 4. Selected bacteria

Names and taxIDs obtained from the NCBI Taxonomy database[3].

Genus	Scientific name	TaxID	Taxon level
**Atypical**			
Mycoplasma	Mycoplasma pneumoniae	2104	Species
	Mycoplasma genitalium	2097	Species
**Gram-negative**			
Bordetella	Bordetella pertussis	520	Species
Burkholderia	Burkholderia cepacia	292	Species
Campylobacter	Campylobacter jejuni	197	Species
Chlamydia	Chlamydia trachomatis	813	Species
Escherichia	Escherichia coli	562	Species
Haemophilus	Haemophilus influenzae	727	Species
Klebsiella	Klebsiella pneumoniae	573	Species
Legionella	Legionella pneumophila	446	Species
Neisseria	Neisseria gonorrhoeae	485	Species
Proteus	Proteus mirabilis	584	Species
Pseudomonas	Pseudomonas aeruginosa	287	Species
Salmonella	Salmonella enterica subsp. enterica serovar Typhimurium	90371	Serovar
	Salmonella enterica subsp. enterica serovar Enteritidis	149539	Serovar
Shigella	Shigella flexneri	623	Species
	Shigella boydii	621	Species
	Shigella dysenteriae*	622	Species
**Gram-positive**			
Bacillus	Bacillus cereus	1396	Species
Clostridium	Clostridium tetani	1513	Species
	Clostridium difficile*	1496	Species
Corynebacterium	Corynebacterium diphtheriae	1717	Species
Staphylococcus	Staphylococcus aureus	1280	Species
	Staphylococcus epidermidis	1282	Species
Streptococcus	Streptococcus pneumoniae	1313	Species
	Streptococcus pyogenes	1314	Species

*Subspecies were included by necessity

Supplementary Table 5. Selected parasites

Names and taxIDs obtained from the NCBI Taxonomy database[3].

Genus	Scientific name	TaxID	Taxon level
**Parasites**			
Trichinella	Trichinella spiralis	6334	Species
Trichomonas	Trichomonas vaginalis	5722	Species

Supplement Table 6. Used HLA-I molecules

Allele frequencies for Europe were obtained from the Allele Frequency Net Database[4].

Locus	Allele	% individuals with allele
**A**		
	A*02:01	15.08
	A*01:01	10.28
	A*03:01	8.68
	A*24:02	6.14
	A*11:02	3.80
**B**		
	B*08:01	6.77
	B*07:02	6.02
	B*44:02	5.48
	B*51:01	4.89
	B*35:01	4.73
**C**		
	C*07:01	11.58
	C*07:02	8.21
	C*04:01	7.80
	C*05:01	6.09
	C*06:02	5.46

Supplementary Table 7. Used HLA-II molecules

Allele frequencies for Europe were obtained from the Allele Frequency Net Database[4].

Locus	Allele	% individuals with allele
**DPA1**		
	DPA1*01:03	19.40
	DPA1*02:01	6.60
	DPA1*02:02	1.04
	DPA1*04:01	0.60
	DPA1*01:04	0.50
**DPB1**		
	DPB1*04:01	27.63
	DPB1*02:01	13.17
	DPB1*04:02	10.19
	DPB1*03:01	7.60
	DPB1*107:01	6.50
**DQA1**		
	DQA1*05:05	25.51
	DQA1*05:01	12.92
	DQA1*01:02	10.91
	DQA1*03:01	8.25
	DQA1*02:01	6.61
**DQB1**		
	DQB1*03:01	12.81
	DQB1*02:01	9.67
	DQB1*05:01	6.50
	DQB1*06:02	6.17
	DQB1*02:02	5.71
**DRB1**		
	DRB1*15:01	6.67
	DRB1*07:01	6.65
	DRB1*03:01	6.25
	DRB1*11:01	4.46
	DRB1*01:01	4.31

Supplementary Figures

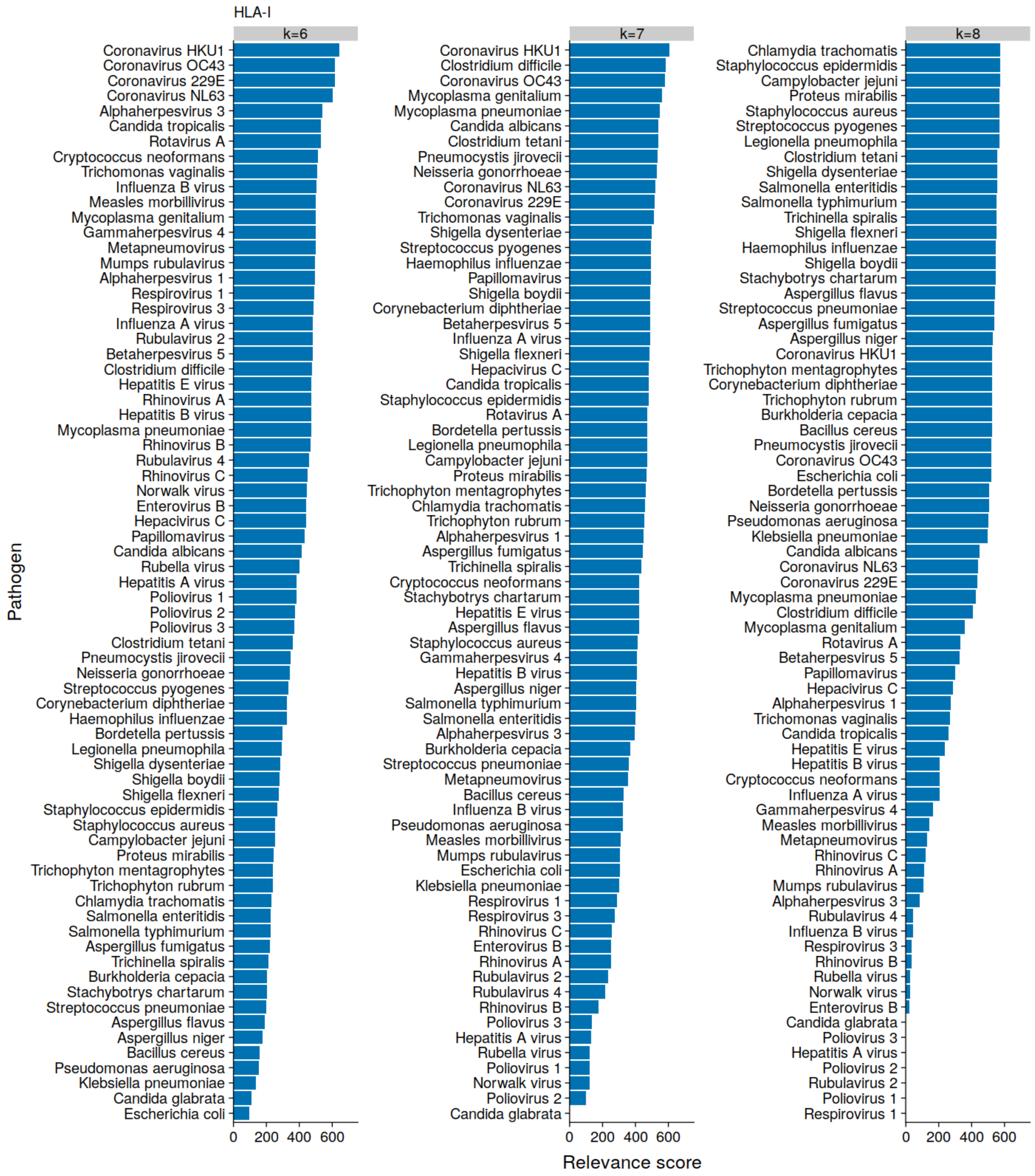

Supplementary Figure 1: Pathogen relevance score for HLA-I SARS-CoV-2 epitopes

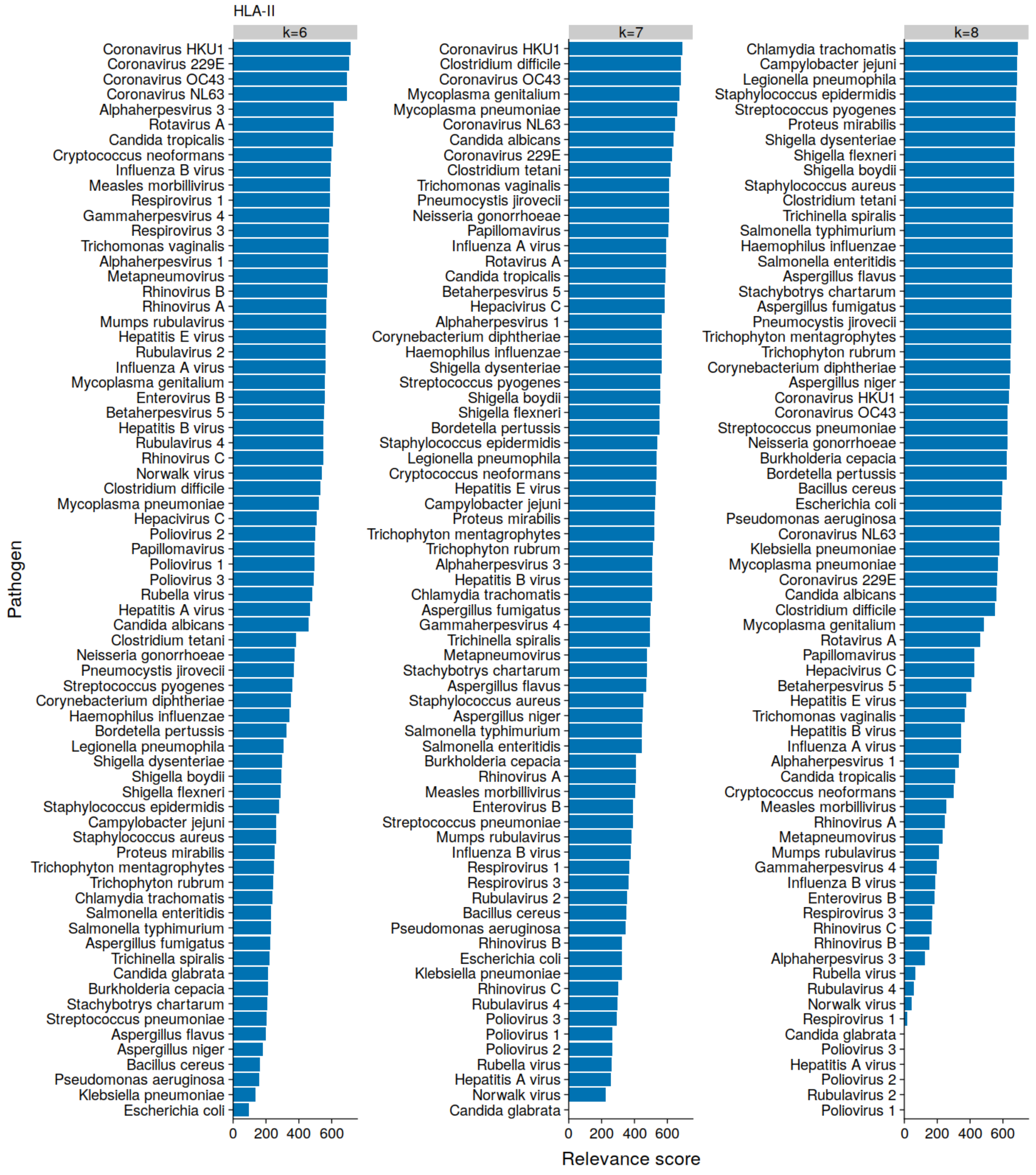

Supplementary Figure 2: Pathogen relevance score for HLA-II SARS-CoV-2 epitopes

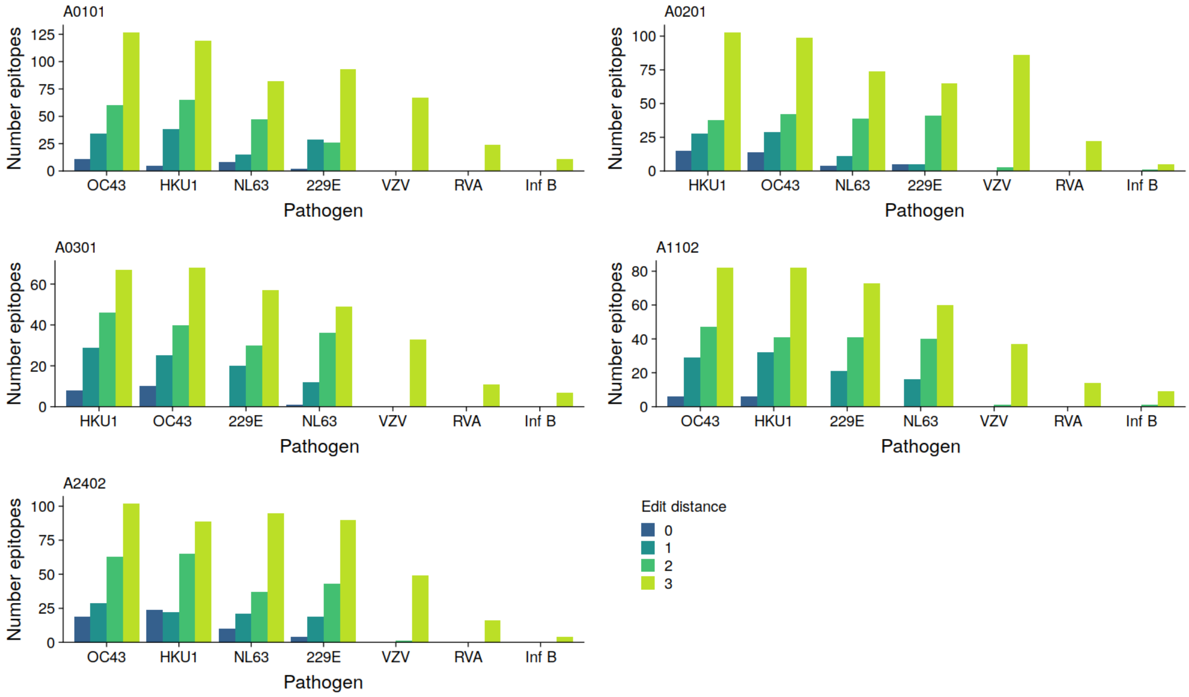

Supplementary Figure 3: Edit distance between HLA-A SARS-CoV-2 epitopes and epitopes in selected pathogen

The Levenshtein distance is here taken as the edit distance.

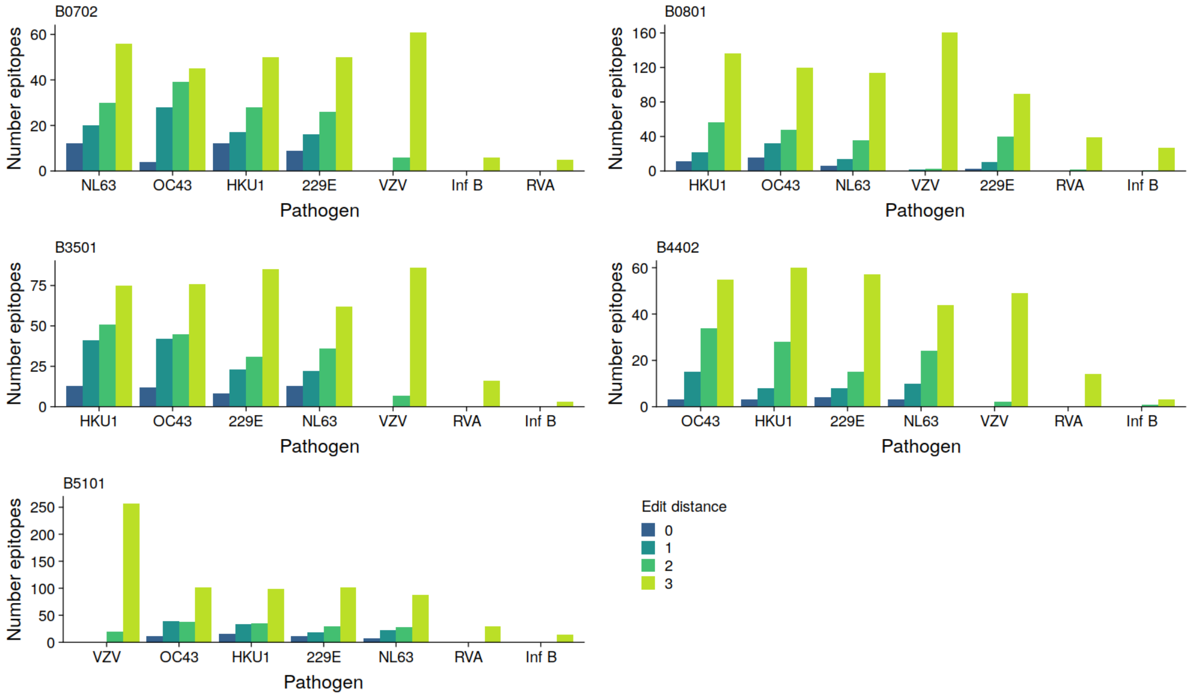

Supplementary Figure 4: Edit distance between HLA-B SARS-CoV-2 epitopes and epitopes in selected pathogen

The Levenshtein distance is here taken as the edit distance.

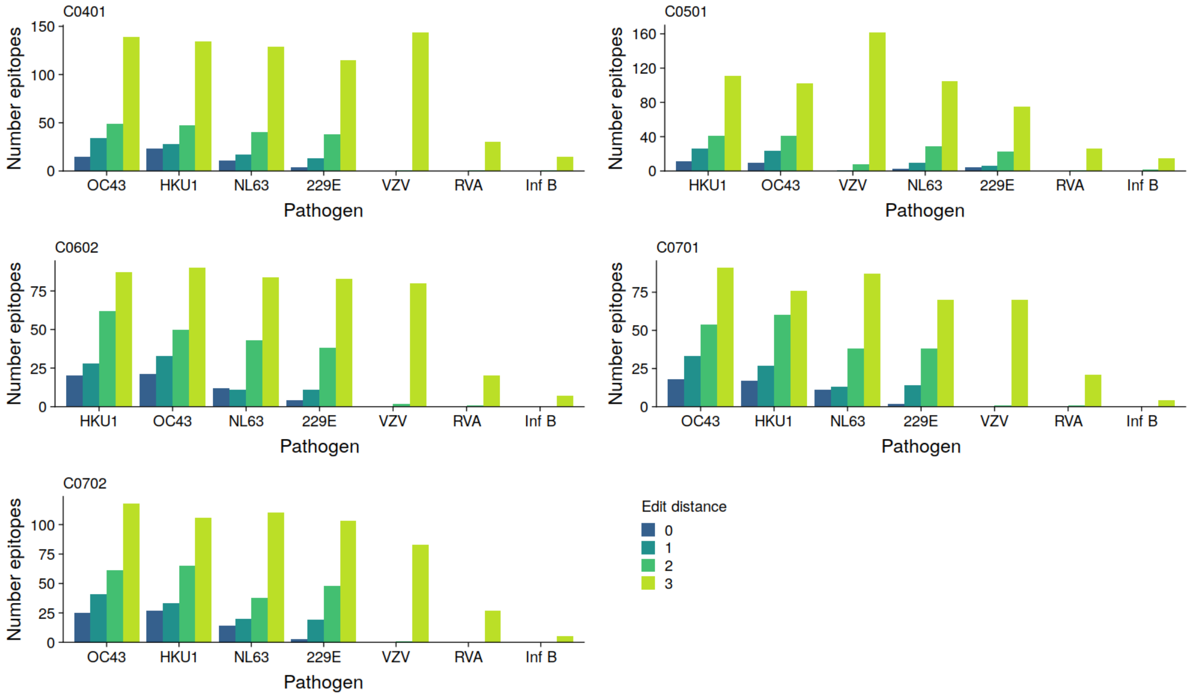

Supplementary Figure 5: Edit distance between HLA-C SARS-CoV-2 epitopes and epitopes in selected pathogen

The Levenshtein distance is here taken as the edit distance.

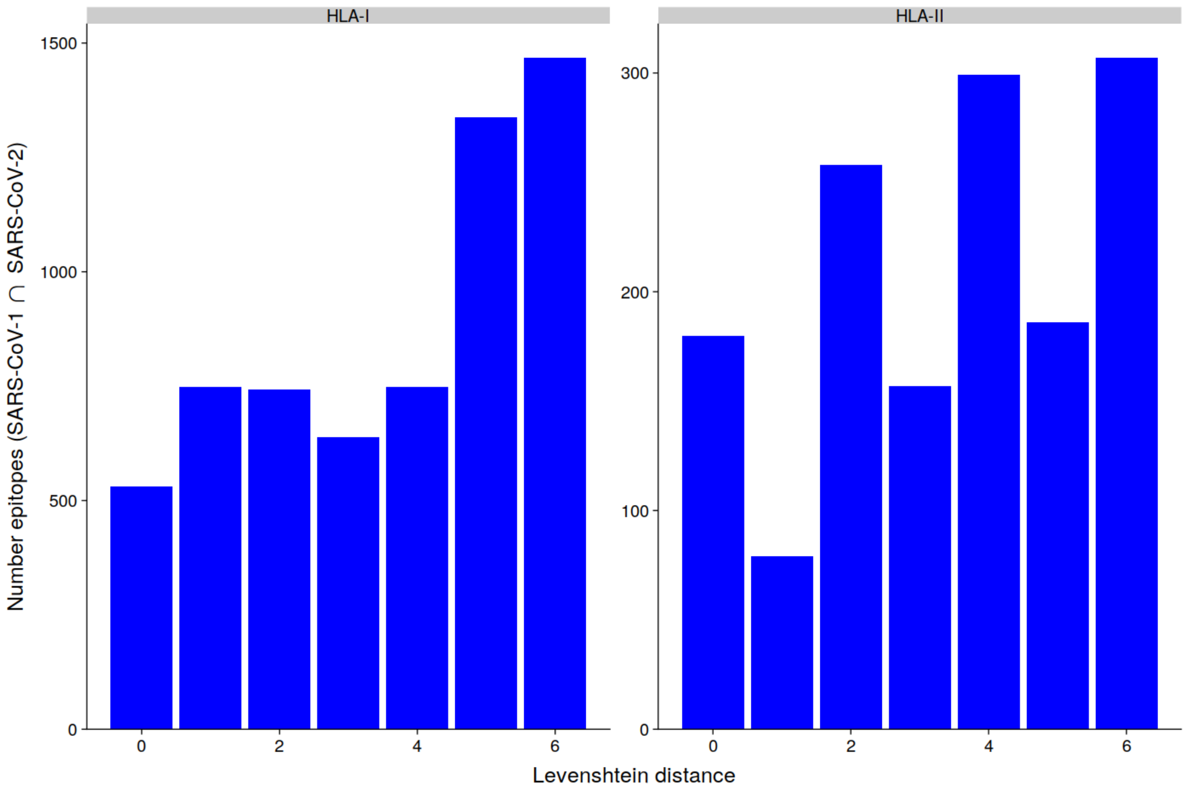

Supplementary Figure 6: Number of known epitopes SARS-CoV-1 shared with predicted SARS-CoV-2 epitopes.

The known SARS-CoV-1 were obtained from iedb.org and compared to the predicted SARS-CoV-2 epitopes using the edit distance. The Levenshtein distance is here taken as the edit distance.

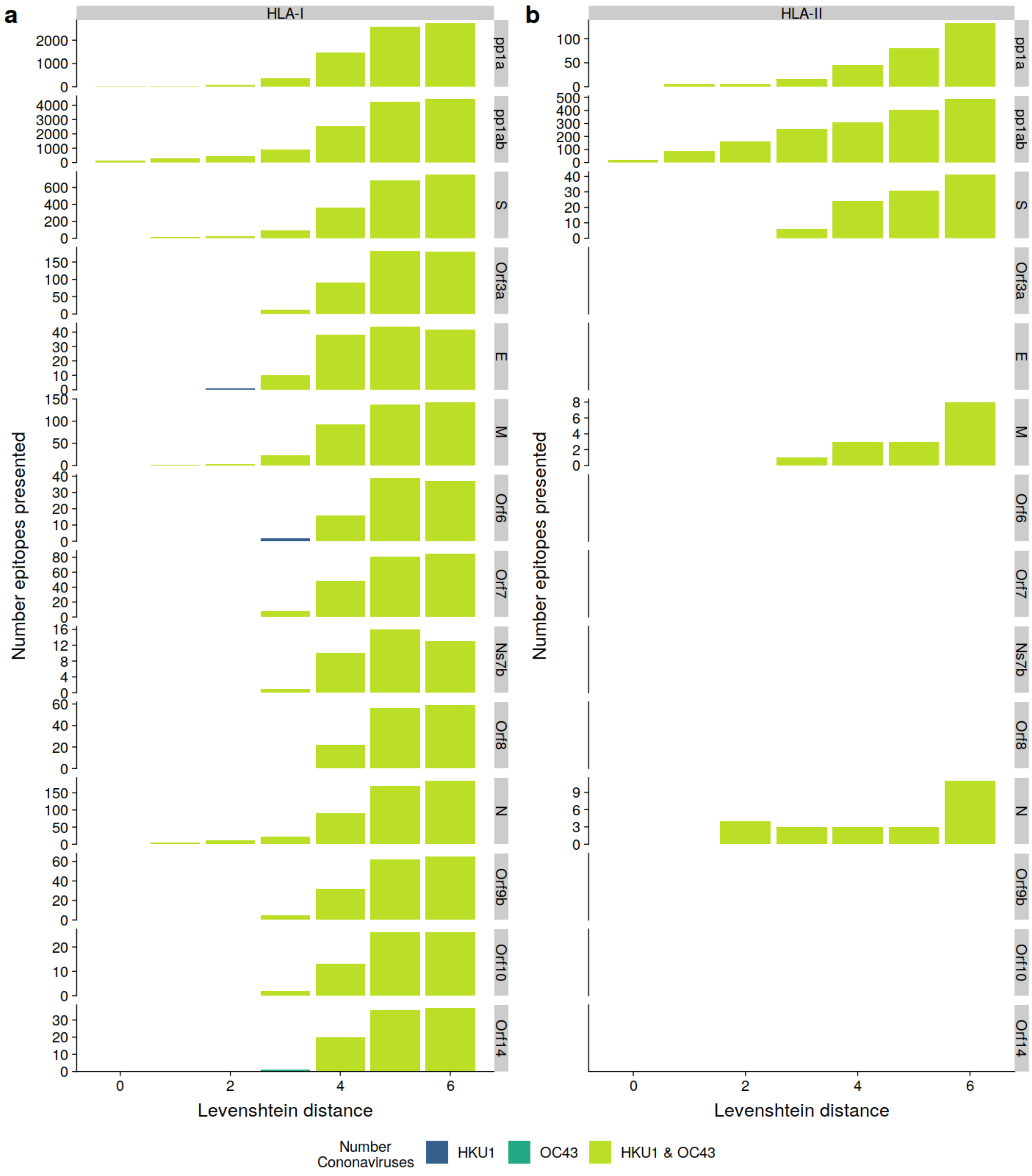

Supplementary Figure 7: Number of HLA-I and HLA-II presented epitopes in each SARS-CoV-2 protein

The number of SARS-CoV-2 epitopes are compared to the epitopes predicted in HKU1 and OC43 using the edit distance. Only HLA-II presented epitopes from pp1a, pp1ab, S, M, and N proteins had an edit distance of 6 or less. The Levenshtein distance is here taken as the edit distance.

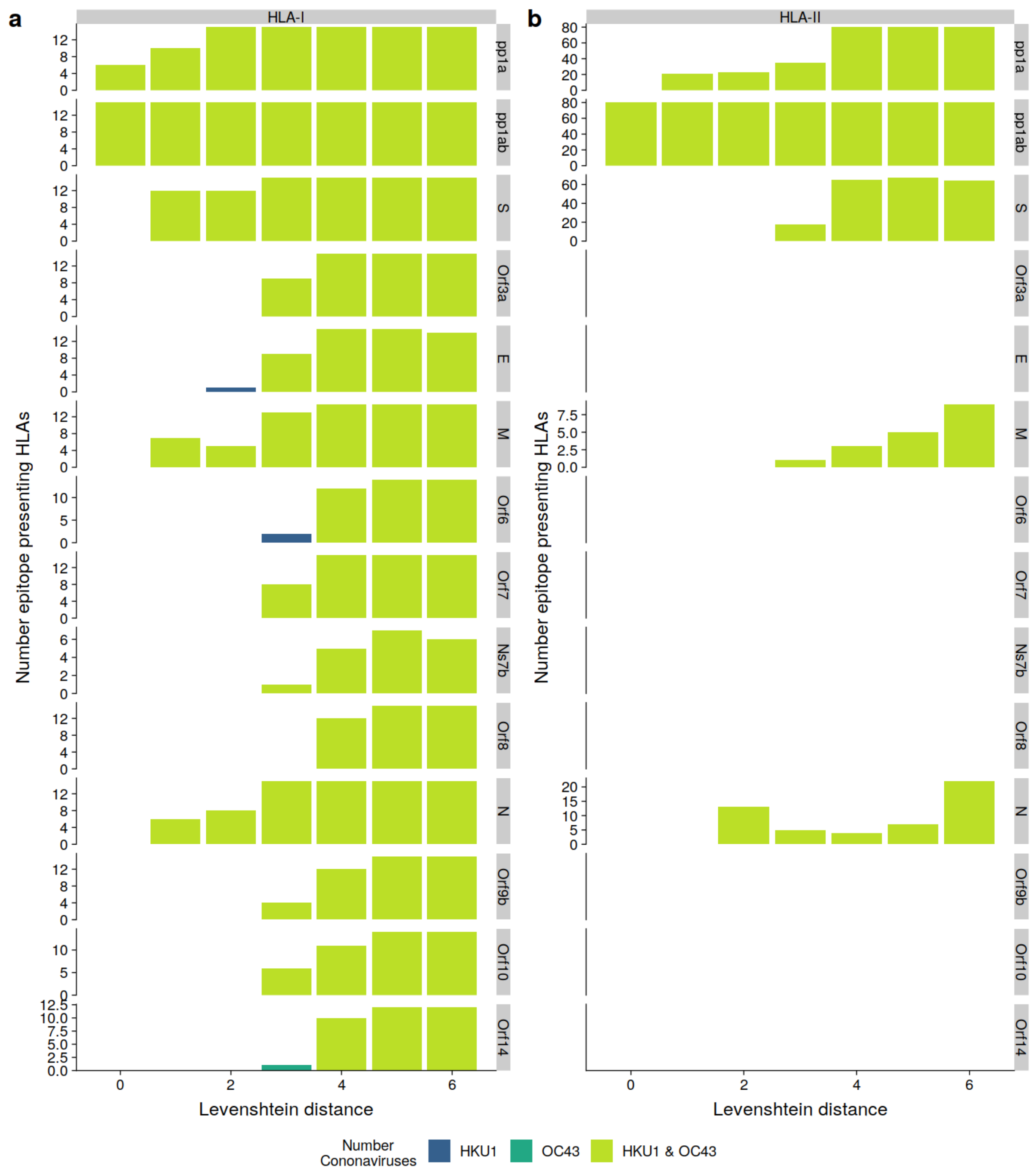

Supplementary Figure 8: Number of different epitopes presenting HLAs identified for epitope in each SARS-CoV-2 protein.

The highest number of possible HLAs are 15 for HLA-I and 80 for HLA-II (50 DPA1-DPB1 combinations, 25 DQA1-DQB1 combinations, and 5 DRB1). Only HLA-II presented epitopes from pp1a, pp1ab, S, M, and N proteins had a edit distance of 6 or less. The Levenshtein distance is here taken as the edit distance.

References

1. Koster, J. & Rahmann, S. Snakemake--a scalable bioinformatics workflow engine. *Bioinformatics* **28**, 2520–2522 (2012).
2. Hulo, C. *et al.* ViralZone: a knowledge resource to understand virus diversity. *Nucleic Acids Res.* **39**, D576–D582 (2011).
3. Federhen, S. The NCBI Taxonomy database. *Nucleic Acids Res.* **40**, D136–D143 (2012).
4. Gonzalez-Galarza, F. F. *et al.* Allele frequency net database (AFND) 2020 update: gold-standard data classification, open access genotype data and new query tools. *Nucleic Acids Res.* **48**, D783–D788 (2020).
